# Alignment of menu items offered in Canadian long-term care homes with Canada’s food guide and the Diabetes Canada Clinical Practice Guidelines

**DOI:** 10.1186/s12889-025-21803-7

**Published:** 2025-02-13

**Authors:** Caroline G. Middleton, Jennifer J. Lee, Mary R. L’Abbé

**Affiliations:** 1 Department of Nutritional Sciences, Temerty Faculty of Medicine, University of Toronto, Toronto, ON Canada; 2 School of Nutrition, Toronto Metropolitan University, Toronto, ON Canada

**Keywords:** Long-term care, Canada’s food guide, Diabetes, Dietary intake, Aging

## Abstract

**Background:**

Many residents in long-term care (LTC) homes face the risk of malnutrition and non-communicable diseases like diabetes, underscoring the crucial role of menu planning. In most provinces, menu items offered in LTC homes must adhere to Canada’s food guide (CFG). Other dietary guidelines, like those in Diabetes Canada Clinical Practice Guidelines (DCCP), provide recommendations for managing chronic disease; however, the alignment of individual menu items with CFG and DCCP is unknown. The objective was to assess the alignment of menu items offered in LTC homes with CFG and DCCP.

**Methods:**

Using a four-week menu cycle designed for LTC, menu items (*n* = 1,365) were assessed using two nutrient profile models based on CFG and the DCCP. The Canadian Foods Scoring System (CFSS) categorized items as “very poor” to “excellent” choices according to CFG, and the DCCP nutrient profile model classified items as “least” to “mostly aligned” with DCCP. Descriptive statistics summarized menu items by CFSS and DCCP nutrient profile model categories across meal occasions and food categories.

**Results:**

Overall, 52.8% of menu items served in LTC homes were rated “good” or “excellent” choices by CFSS, and 50.8% were classified as “most aligned” with the DCCP nutrient profile model. Afternoon Snacks had the highest proportion of the least healthy items. *Legumes* and *Vegetables* were the healthiest categories, and *Sugars & Sweets*, along with *Combination Dishes*, ranked as the least healthy.

**Conclusions:**

While about one-half of LTC menu items align with CFG and DCCP, opportunities remain to enhance their nutritional quality. Developing a translational tool based on nutrient profile models could simplify the application of food-based dietary guidelines, supporting more effective and aligned LTC menu planning.

## Background

Over 400,000 Canadians currently live in long-term care (LTC) homes (e.g., nursing homes, care homes, and skilled nursing facilities), which provide essential 24-h nursing care and support for daily living activities and healthcare needs [1]. Poor nutrition is a significant concern for older adults, increasing their risk of age-related malnutrition and non-communicable diseases [2, 3]. Malnutrition affects up to 60% of LTC residents, with 28.9% to 53.7% of older adults in Canadian LTC homes identified as malnourished or at risk, depending on the measurement tool used [4, 5]. Nearly three-quarters of adults over 65 have at least one of the top ten non-communicable diseases [6]. Diabetes, a leading cause of death, raises the risk of other diseases like cardiovascular diseases, chronic kidney diseases, and cancers [7]. Approximately 25% of LTC residents have diabetes, often accompanied by cardiovascular complications and substantial comorbidity [8]. These statistics underscore the critical role of health management, dietary planning, and nutritional support in addressing the high prevalence of malnutrition and chronic diseases in LTC. Furthermore, the food environment in LTC homes is integral to the overall quality of life and health of residents.

Several healthy eating guidelines and dietary recommendations currently exist to support healthy eating among Canadians in LTC. While not required in all provinces, most Canadian jurisdictions require that Registered Dietitians or Nutrition Managers develop or review LTC menus to ensure alignment with Canada’s food guide (CFG) [9], Dietary Reference Intakes [10], and provincial regulations and standards [11]. The CFG, revised in 2019, emphasizes the consumption of vegetables and fruits, whole grain foods, and lean protein foods, including plant-based protein foods [9]. However, the lack of specific serving sizes or quantitative goals in CFG 2019 poses challenges for LTC homes attempting to adhere to these recommendations for health and disease management [11, 12]. Consumption patterns indicate that protein foods account for 33.4% of daily caloric intake (31.6% animal-based, 1.8% plant-based), while vegetables and fruits represent only 10.3%, with the majority being prepared with additions like salt or sugar [13]. Other foods (food groups not recommended in CFG), including juice and desserts, contribute 31.3% of daily calories, further highlighting an imbalance in menu offerings [13]. These findings highlight the need for improved strategies to better align LTC menus with dietary guidelines and address residents’ nutritional needs.

Diabetes Canada has published dietary recommendations in the Diabetes Canada Clinical Practice Guidelines (DCCP) to help Canadians with or at risk for diabetes manage their condition, including pre-diabetes and diabetes [14]. Unlike the broader focus of CFG on preventing non-communicable diseases, the DCCP include targeted recommendations for diabetes management, such as emphasizing foods with a lower glycemic index and setting specific thresholds for nutrients of concern, like added sugars and saturated fat [14]. However, these guidelines are not specifically tailored to the unique dietary needs of older adults commonly found in LTC homes [14]. Given the high prevalence of chronic diseases, including diabetes, in LTC, incorporating flexible dietary principles aligned with these recommendations can support chronic disease management and improve residents’ quality of life [8].

Therefore, the objective of the study was to examine the alignment of menu items offered in LTC homes with the dietary recommendations of CFG 2019 and the DCCP.

## Methods

### LTC menu planning considerations

LTC homes provide housing, care, and meals to older adults (defined as those aged 65 years or older), with an average resident age of 83 years [15]. These homes must offer three meals and at least two snacks daily, with each meal typically including a choice between two entrée options—commonly one hot and one cold—to accommodate individual resident preferences [11]. While some LTC homes prepare meals in-house, many rely on external food service providers that supply pre-planned menus. These menus typically follow a 21- to 28-day cycle structure, with periodic reviews and updates incorporating seasonal changes for food availability and multiple options to meet residents’ needs and preferences [11].

Food in LTC serves both nutritional and functional purposes, supporting residents’ dietary needs and contributing to their overall well-being [15]. To address the prevalence of malnutrition in LTC homes, general Canadian guidelines recommend the liberalization of therapeutic diets to encourage food intake, as restrictive diets are associated with reduced consumption and malnutrition risk [11, 15]. Menu planning aims to balance nutritional adequacy with cultural and individual preferences, ensuring that menus include nutrient-dense, easily consumed foods to address barriers such as poor oral health, sensory changes, early satiety, and fatigue [11]. Nutrient analysis is recommended to ensure that menus meet daily nutritional targets, comply with regulatory requirements, and support the health and quality of life of LTC residents [16].

### LTC menu item database

This study analyzed a menu database containing menu and nutrient information for the Spring/Summer 2023 four-week menu cycle, designed for individuals aged 70 years and older following a regular diet (i.e., a standardized diet without specific dietary restrictions or modifications). The database was sourced from a leading North American food service provider that provides sample menu cycles to a wide range of LTC homes in Canada and elsewhere, including nursing homes, care homes, and skilled nursing facilities. The database included information on the nutritional composition, ingredients, portion sizes, and meal occasions (breakfast, lunch, dinner, and snacks). Nutritional profiles were derived by the food service provider from GS1 standards (Brussels, Belgium), with any missing nutrient values supplemented by our group using the Canadian Nutrient File database (2015), a standard reference food composition database maintained by Health Canada that contains nutrient information of foods commonly consumed in Canada [17]. The four-week menu cycle consisted of 1,365 menu items, including foods (*n* = 532), recipes (*n* = 392), and beverages (*n* = 441). Duplicates (*n* = 1,087) were included in the analysis, as this study examined all menu items offered. Menu items were categorized using Health Canada’s Table of Reference Amounts for Food [18], which represents the amount of food typically consumed in one sitting and serves as the basis for determining serving sizes (i.e., reference amount) for pre-packaged foods in the Nutrition Facts table.

### Nutrient profile models

Nutrient profile models were used to assess the healthfulness of menu items in the LTC menu database according to the dietary recommendations in CFG 2019 and the DCCP. A nutrient profile model classifies or ranks foods according to their nutritional composition for reasons related to preventing disease and promoting health [19].

#### Canadian food scoring system

The alignment of individual menu items with CFG was assessed using the Canadian Food Scoring System (CFSS), which is described in detail elsewhere [20]. Briefly, foods (i.e., single-ingredient foods, multi-ingredient foods, and recipes) and beverages were categorized and assigned points based on Canada’s Dietary Guidelines [21], detailed dietary recommendations in which CFG recommendations are based on. Deduction proportions were then applied based on the levels of three nutrients-of-concern (i.e., saturated fat, sodium, and sugars), recommended to limit according to Canada’s Dietary Guidelines [21]. The final score, ranging from 10 to 100, with higher scores indicating a “healthier” choice, was then calculated to categorize menu items into one of five CFSS categories: “very poor (CFSS score range: 10–29),” “poor (30–49),” “fair (50–69),” “good (70–89),” or “excellent (90–100)” choice [20].

#### DCCP nutrient profile model

The DCCP nutrient profile model assesses individual menu items for alignment with the DCCP [14], assigning points to foods based on macronutrient or meal quality, saturated and trans-fat content, and added sugar content, and to beverages based on beverage type, saturated fat, and added sugar content [22]. Points from each step are counted and converted, when necessary, into a scale of 0 to 6. The final score was used to classify products into one of three categories for their alignment with the DCCP: ‘Least Aligned’ (e.g., 0–2 points), ‘Partially Aligned’ (e.g., 2.5–4 points), and ‘Most Aligned’ (e.g., 4.5–6 points) [22]. A higher score indicates better alignment with the DCCP or a “healthier” item. Since the DCCP nutrient profile model focuses on ingredients and proportions, it does not require modification for recipes, as they can be classified as *Combination Dishes*, according to Health Canada’s Table of Reference Amounts [18].

### Statistical analyses

Descriptive statistics, presented as the proportion of menu items (n, %), were used to summarize Canadian LTC menu items. All menu items were categorized by food group and meal occasion. Following CFG 2019 recommendations [9], food grouping categories include Fruits & Vegetables (e.g., fresh, frozen, or canned options aligned with CFG recommendations, excluding those with added sugars or sodium), Protein Foods (e.g., plant-based options like legumes, nuts, seeds, and animal-based sources like lean meats, fish, eggs, and dairy), Whole Grain Foods (e.g., whole grain bread, oatmeal, brown rice), Other Foods (e.g., refined grains, sweets, sweetened beverages), and Water (e.g., plain water, naturally flavored water, unsweetened coffee, tea), distributed across six daily meal occasions: Breakfast, Morning Snack, Lunch, Afternoon Snack, Dinner, and Evening Snack. Menu items were further categorized based on the CFSS and DCCP nutrient profile model scores overall and across these meal occasions and aligned with the Table of Reference Amounts for Food major categories [18]. All analyses were performed using R statistical software (Version 4.3.2).

## Results

### Database characteristics

Menu items offered varied by meal occasion, with Breakfast and Lunch offering the highest number of items (*n* = 254 each) and Morning Snacks the fewest (*n* = 56) (Table 1). Fruits & Vegetables were most prevalent in Afternoon Snacks (33.3%, *n* = 28/84), including grapes and apple slices. Protein Foods were less common at Breakfast (4.8%, *n* = 4/84) but were more common in Morning Snacks (50.0%, *n* = 28/56) and Evening Snacks (42.4%, *n* = 36/85), featuring unsweetened milk and yogurt. Whole Grain Foods were most frequently included at Breakfast (22.0%, *n* = 56/254) with items like whole-grain toast. Other Foods varied widely across meal occasions, with the highest percentages observed at Lunch (28.7%, *n* = 73/254) and Afternoon Snacks (26.4%, *n* = 24/84), often including cookies or pudding. Water was consistently available across all meal occasions.

**Table 1:** Long-term care homes’ menu composition by food category and meal occasion

Meal occasion	Overall	Food categories				
		**Fruits & vegetables**	**Protein foods**	**Whole grain foods**	**Other foods**	**Water**
Breakfast	254	58 (22.8%)	56 (22.0%)	56 (22.0%)	56 (22.0%)	28 (11.0%)
Morning Snack	56	0 (0.0%)	28 (50.0%)	0 (0.0%)	0 (0.0%)	28 (50.0%)
Lunch	254	77 (30.3%)	67 (26.4%)	9 (3.5%)	73 (28.7%)	28 (11.0%)
Afternoon Snack	84	28 (33.3%)	4 (4.8%)	0 (0.0%)	24 (28.6%)	28 (33.3%)
Dinner	231	57 (24.7%)	58 (25.1%)	27 (11.7%)	61 (26.4%)	28 (12.1%)
Evening Snack	85	1 (1.2%)	36 (42.4%)	12 (14.1%)	8 (9.4%)	28 (32.9%)

The proportion of menu items (*n*, %) across Canada’s food guide 2019 food categories (i.e., Fruits & vegetables, Protein foods, Whole grain foods, Other foods, and Water) is shown overall and by meal occasion (i.e., Breakfast, Morning Snack, Lunch, Afternoon Snack, Dinner, and Evening Snack)

### Characterizing LTC menu items using CFSS

**Fig. 1:**
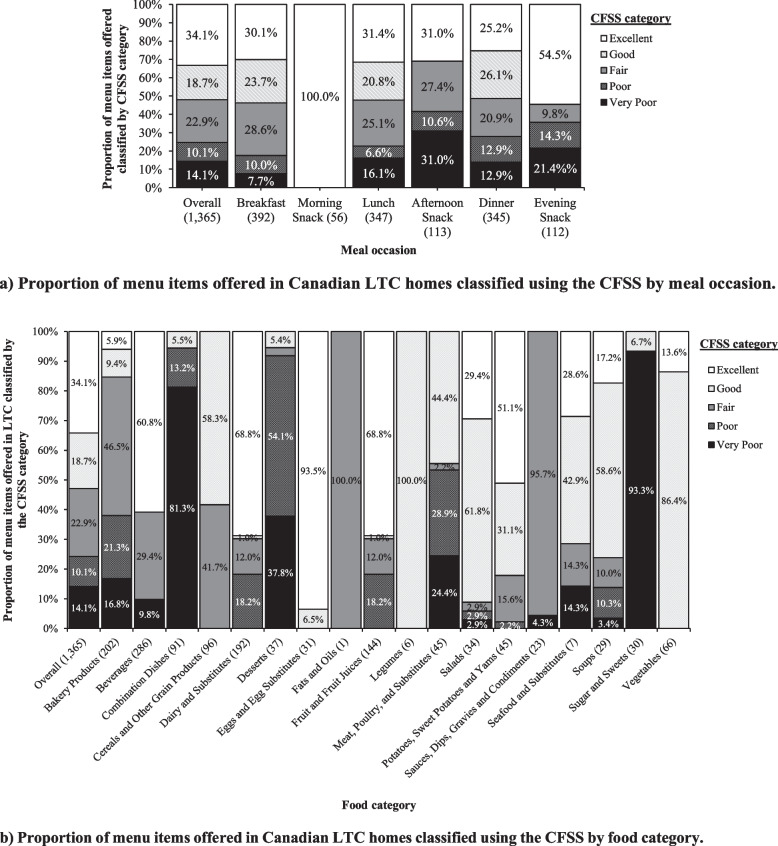
**a** Proportions shown overall and by meal occasion (i.e., Breakfast, Morning Snack, Lunch, Afternoon Snack, Dinner, and Evening Snack); **b** Proportions shown overall and by Table of Reference Amounts for Food major category [18] in alphabetical order. Sample sizes (*n*) for each food category shown in parentheses. The CFSS classified menu items into one of five categories: “Excellent,” “Good,” “Fair,” “Poor,” or “Very Poor” choices according to their alignment with Canada’s food guide 2019. Abbreviations: CFSS, Canadian Food Scoring System; LTC, long-term care

Overall, 34.1% of menu items (*n* = 466/1,365) were classified as “excellent,” 18.7% (*n* = 255/1,365) as “good,” 22.9% (*n* = 313/1,365) as “fair,” 10.1% (*n* = 138/1,365) as “poor,” and 14.1% (*n* = 193/1,365) as “very poor” choice. Figure 1a shows the proportion of menu items (n, %) across CFSS categories (i.e., “very poor,” “poor,” “fair,” “good,” and “excellent”) overall and by meal occasion. Across meal occasions, Breakfast (53.8%), Morning Snacks (100%), Lunch (52.2%), Dinner (51.3%), and Evening Snacks (54.5%) had over one-half of the menu items classified as either “good” or “excellent,” according to CFSS. In contrast, Afternoon Snacks had the highest proportion of menu items scoring “very poor” or “poor” with CFSS, at 10.6% and 31.0%, respectively. Food categories with the highest proportion (> 80%) of menu items classified as “good” or “excellent” were *Eggs & Egg Substitutes*, *Vegetables*, *Legumes*, *Salads*, and *Potatoes, Sweet Potatoes, & Yams* (Fig. 1b). On the other hand, food categories with the highest proportion of menu items classified as “poor” or “very poor” were *Sugars & Sweets, Combination Dishes,* and *Desserts*.

### Characterizing LTC menu items using DCCP nutrient profile model

**Fig. 2:**
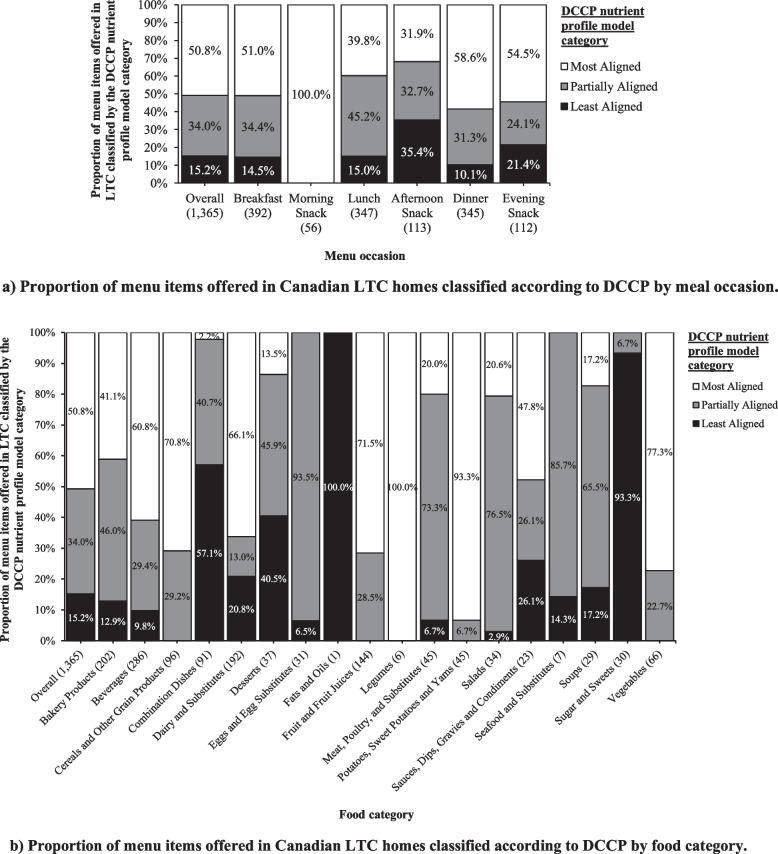
**a** Proportions shown overall and by meal occasion (i.e., Breakfast, Morning Snack, Lunch, Afternoon Snack, Dinner, and Evening Snack); **b** Proportions shown overall and by Table of Reference Amounts for Food major category [18] in alphabetical order. Sample sizes (*n*) for each food category shown in parentheses. The DCCP nutrient profile model classified menu items into one of three categories: “Most Aligned,” “Partially Aligned,” and “Least Aligned,” choice according to Diabetes Canada Clinical Practice Guidelines. Abbreviations: DCCP, Diabetes Canada Clinical Practice Guidelines; LTC, long-term care

Overall, 50.8% of foods (*n* = 693/1,365) were classified as “most aligned,” 34.0% (*n* = 464/1,365) as “partially aligned,” and 15.2% (*n* = 208/1,365) as “least aligned” with the DCCP. While Breakfast (51.0%), Morning Snacks (100%), Dinner (58.6%), and Evening Snacks (54.5%) had over one-half of the menu items scoring “most aligned” with the DCCP, Afternoon Snacks and Lunch scored lower, with only 31.9% and 39.8% of items aligning closely with the DCCP (Fig. 2a). Food categories with the highest proportion of menu items classified as “most aligned” were *Legumes* and *Potatoes* (Fig. 2b). Similarly, food categories with the highest proportion of menu items classified as “partially aligned” were *Eggs & Egg Substitutes* and *Seafood & Substitutes*. However, the food category with the highest proportion of menu items classified as “least aligned” was *Sugars & Sweets*.

## Discussion

This study assessed all menu items offered by a major provider of foods for Canadian LTC, according to the dietary recommendations outlined in CFG and the DCCP. Approximately one-half of the menu items received ratings of “good” or “excellent” according to the CFSS (scoring system based on the CFG recommendations) and were classified as “most aligned” according to the DCCP nutrient profile model. Afternoon Snacks had the highest proportion of poorly rated menu items across all meal occasions, according to the CFSS and DCCP nutrient profile model. Both models identified *Legumes* and *Vegetables* as the healthiest food categories, while *Sugars & Sweets* and *Combination Dishes* were the least healthy. Additionally, less aligned foods were offered more often than healthier options; for example, *Sugar & Sweets* (*n* = 30/1,365) were offered more frequently than *Legumes* (*n* = 6/1,365), indicating the limited availability of nutrient-dense choices.

Menu items in LTC frequently scored lower when they deviated from dietary guidelines, often due to the inclusion of refined grains, high sodium levels, added sugars, and low nutrient density. Similar to previous studies on Canadian LTC menus [13], many menu items were less aligned with CFG 2019 recommendations, largely due to the high consumption of refined grains, less healthful vegetables and fruits often prepared with added sodium or sugar, and a predominance of animal-based proteins—all contrary to CFG recommendations [13]. Lunches, for example, often featured high-sodium soups, refined bread sandwiches, and processed deli meats, while *Combination Dishes* such as casseroles and pasta-based meals relied on refined grains, sodium-rich sauces, and dressings high in saturated fats. Afternoon Snacks, which included cookies, flavored pudding cups, and sugar-sweetened beverages, also contributed to lower scores, lacking essential nutrients and adding excessive sodium and sugars. Replacing Afternoon Snacks with nutrient-dense options like fresh fruits, fortified soy or dairy products, or nut-based snacks, and incorporating whole grains, lower-sodium soups, and lean proteins into lunches could enhance nutrient density. Reformulating *Combination Dishes* with whole grains, lower-sodium sauces, and added vegetables would also support better menu quality. Cultural practices, such as the preference for sweeter snacks in the afternoon, may further inform interventions, emphasizing culturally acceptable but more healthful choices. Addressing these dietary gaps by prioritizing legumes, vegetables, and fortified soy or dairy products across meals would not only better align menus with CFG 2019 but also reduce malnutrition risk and enhance the overall health and well-being of LTC residents.

Menu planning in LTC homes is governed by provincial regulations requiring compliance with CFG and/or Dietary Reference Intakes to ensure dietary quality meets the nutritional needs of residents [11]. While professional oversight by Registered Dietitians and Nutrition Managers provides a robust framework for menu planning, challenges persist in translating guidelines into practice [11]. There is currently no straightforward tool to assess menu alignment with CFG, making it difficult for planners to ensure compliance while addressing diverse resident needs [15]. Unlike the CFG 2007 [23], which provided clear serving sizes and amounts for each food group, the CFG 2019 focuses on whole-day dietary proportions, making it more challenging to translate into specific menu offerings [9]. Additionally, policies that require collaboration with Resident Councils to incorporate preferences, while essential for resident satisfaction and autonomy, may inadvertently prioritize less healthy options over CFG-aligned foods [11]. This need to compromise between regulatory nutritional guidelines and resident preferences reveals a critical gap in the menu planning process. To address these challenges, developing practical tools to assess CFG compliance and offering additional resources for menu planners are essential steps. Such tools could help bridge the gap between meeting regulatory requirements, aligning menus with dietary guidelines, and accommodating resident preferences, ultimately improving the nutritional quality of LTC menus and enhancing the health outcomes of residents.

Nutrient profiling models can be used to design menus that align with nutritional guidelines. Our study highlighted differences between the items’ alignment with CFG and the DCCP. For example, while eggs were rated as “excellent” by the CFSS, they were only “partially aligned” with the DCCP nutrient profile model due to the stricter saturated fat recommendations in the DCCP [14]. This exemplifies the need to consider both systems for comprehensive dietary planning. Using these nutrient profile models in menu planning can ensure that the majority of food items offered in LTC homes meet stringent nutritional standards. Prioritizing foods rated as “excellent” or “good” choices according to CFSS, along with those “most” and “partially aligned” with the DCCP, can help achieve this goal. However, it is also important to include a smaller proportion of preferred foods that may not fully align with these guidelines to enhance resident satisfaction and increase overall food intake [11]. This balanced approach supports the physical and psychological well-being of residents, particularly those malnourished or with limited appetites.

This study examines a comprehensive database of LTC menu items to assess their alignment with dietary guidelines at the meal occasion level. The evaluation uses established frameworks from CFG and the DCCP to identify areas where menu quality can be improved. The findings offer useful insights for future efforts aimed at enhancing the nutritional quality of meals in LTC homes. However, the study has several limitations. First, the study does not assess how menu items align with critical nutritional requirements for residents aged 70 and older, such as calcium, fiber, and vitamin D, despite the high risk of malnutrition in LTC [24, 25]. Future research should be conducted to evaluate menu alignment against nutritional requirements to better address the dietary vulnerabilities of this population. Second, the study evaluated foods offered rather than actual consumption, which may differ significantly in LTC homes, particularly considering that malnutrition affects up to 60% of residents [4]. Such research is needed to provide critical insights into residents’ eating behaviors and inform strategies to refine menu planning, ensuring that the foods provided are both nutritionally adequate and acceptable to residents [26]. Third, the dataset did not account for variations among different types of LTC homes (e.g., nursing homes, skilled nursing facilities, or care homes), and limited research has examined potential differences in their food environments. Although these homes are designed to address the specific needs of their residents, they should all have healthy food environments, and further research in this area could be beneficial to ensure supportive food environments are present regardless of the type of LTC homes. Finally, this study relies on cross-sectional data from a single four-week menu cycle, limiting its ability to capture seasonal or longitudinal variations in menu planning practices.

## Conclusions

Our findings suggest that while many menu items offered in Canadian LTC homes align with CFG and DCCP recommendations, improvements can be made to better adhere to food-based dietary guidelines. While nutrient profile models like the CFSS and the DCCP are valuable for assessing food quality and guiding substitutions to improve menu items, additional translational tools are needed to support LTC menu planning, particularly since CFG and DCCP are challenging to apply directly, and nutrient analysis software is not widely available or used in all LTC homes. Future research should focus on developing tools to effectively implement dietary guidelines, thus improving food quality and adherence to standards in LTC.

## Data Availability

The data analyzed during the current study are available upon reasonable request. For more information on the process for approval to access this data, contact Dr. Mary R. L’Abbé (mary.labbe@utoronto.ca).

## Abbreviations

CFG: Canada’s food guide
CFSS: Canadian Food Scoring System
DCCP: Diabetes Canada Clinical Practice Guidelines
LTC: Long-term care
